# Electro-characteristics of Myocardial Pouches and Reduction of the Frequency of Steam Pops During Radiofrequency Ablation

**DOI:** 10.3389/fphys.2022.816865

**Published:** 2022-01-25

**Authors:** Jianfeng Luo, Fei Guo, Hongjun Zhu, Hao Su, Yuanbo Wu, Jing Zhu, Can Zhang, Jian Xu

**Affiliations:** ^1^Department of Cardiology, The First Affiliated Hospital of USTC, Division of Life Sciences and Medicine, University of Science and Technology of China, Hefei, China; ^2^Department of Neurology, The First Affiliated Hospital of USTC, Division of Life Sciences and Medicine, University of Science and Technology of China, Hefei, China

**Keywords:** arrhythmia, atrial fibrillation, radiofrequency ablation, impedance, pouch, steam pop

## Abstract

**Background**: Radiofrequency ablation (RFA) effectively treats arrhythmia. Steam pop (SP) is a dangerous complication of RFA, which can lead to pericardial tamponade or even death.

**Objective**: This study aimed to explore the electro-characteristics of myocardial pouches, and the relationship between SP, pouch, and impedance.

**Methods**: Swine myocardium was divided into the pouch group and smooth myocardium group. Continuous RFA at 50 W was applied. The initial impedance reduction within the first 3 s of ablation and the time from the start of ablation to SP were recorded. After enabling the delta impedance cutoff function, RFA was performed at different percentage of delta impedance (PDI) cutoff thresholds.

**Results**: The impedance was higher for the pouch myocardium compared to the smooth myocardium (123.22 ± 8.63 Ω and 95.75 ± 4.75 Ω, respectively; *p* < 0.001). The RFA duration before SPs was shorter in the pouch group compared to the smooth myocardium group [9 s (interquartile range, IQR: 6.25–13 s) and 33 s (IQR: 26.25–40.75 s), respectively; *p* < 0.001]. Within the first 3 s of RFA, impedance reduction (24.65 ± 6.57 Ω and 12.78 ± 3.35 Ω, respectively; *p* < 0.001) and PDI [19.18% (IQR: 16.39–24.20%) and 12.96% (IQR: 11.17–14.39%), respectively; *p* < 0.001] were greater in the pouch group compared to the smooth myocardium group. A PDI of 15% and delta time of 3 s effectively reduced the frequency of SPs without seriously affecting RFA use.

**Conclusion**: SPs occur more frequently in the pouch area during RFA. Appropriate delta impedance cutoff settings (PDI: 15%; delta time: 3 s) can reduce the frequency of SPs and improve the RFA safety.

## Introduction

Radiofrequency ablation (RFA) is an effective treatment for patients with arrhythmia (Hindricks et al., 2021). However, there is concern regarding the safety of RFA, especially with the recently introduced high-power, short-time RFA and left atrial isolation (Eichenlaub et al., 2021; Zedda et al., 2021).

During RFA, coagulation necrosis begins at temperatures higher than 50°C. As the tissue temperatures rise, steam is formed within the myocardium, leading to a steam pop (SP). SP refers to the audible sound produced by an intramyocardial explosion when the intra-tissue steam is rapidly produced, causing pressure buildup (Nakagawa et al., 1998). SPs are relatively infrequent (0.1–1.5%) but are a potentially severe complication of RFA; SPs may result in myocardial wall disruption and increased risk for cardiac perforation, pericardial tamponade, embolic stroke, and even death (Seiler et al., 2008; Tokuda et al., 2011). It is difficult to achieve the optimal balance between sufficient energy to penetrate deep myocardial tissues and avoiding excessive heat and SPs. Therefore, it will be useful to develop technology that prevents this potentially life-threatening complication (Kondo et al., 2017; Viles-Gonzalez et al., 2017).

SPs often occur after the changes in myocardial tissue impedance (Theis et al., 2015; Alfonso-Almazán et al., 2019). During RFA, the impedance gradually decreases, sometimes with a steep decrease at the very beginning (Ikeda et al., 2014; Viles-Gonzalez et al., 2017). SP is associated with more rapid impedance reduction at the initial stage (Cooper et al., 2004; Theis et al., 2015; Nguyen et al., 2018). RFA may be interrupted due to impedance changes, based on several important principles. First, the application of radiofrequency produces lesions through resistive heat, which depends on tissue electrical resistivity and is inversely related to tissue water content. Second, tissue impedance initially decreases due to increased mobility of ions. Third, thermal lesions are associated with decreased impedance, and the magnitude of the decrease depends on tissue temperature and area of the heated cardiac tissue. Thus, higher tissue temperature and faster rise in temperature correlate with faster and earlier decrease in impedance. Finally, a rapid increase in temperature (resulting in a rapid and significant decrease in impedance) produces gas from the ablated tissue through vaporization.

If impedance is rapidly reducing or has reduced to a great, it is reasonable to interrupt RFA. However, the cutoff values to guide this decision and prevent SPs have not been determined.

**Figure 1:**
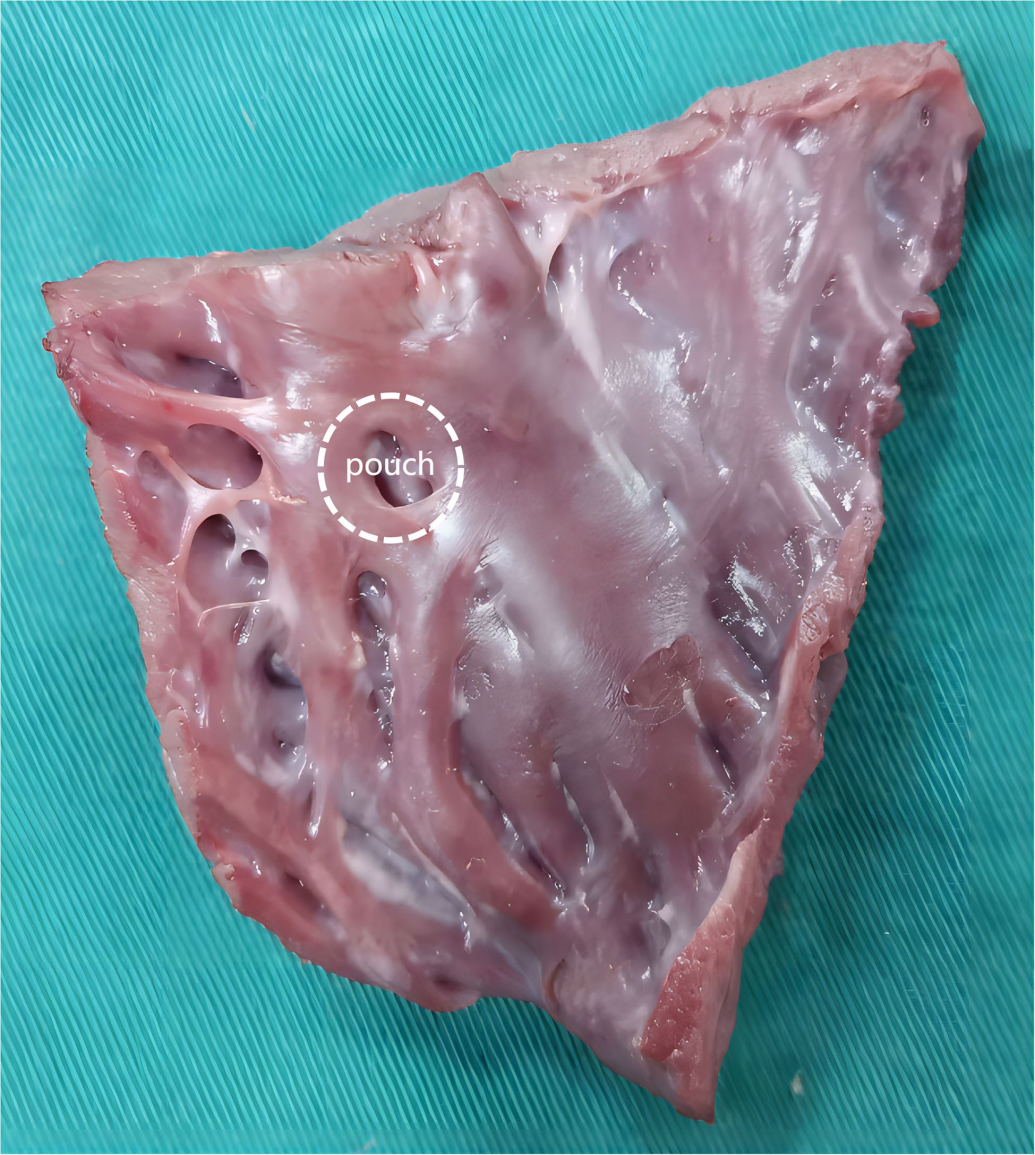
A figure to show pouch.

There are many pocket-shaped indentations of various sizes in the human endocardium, called pouches (Figure 1; Costa et al., 2004; Shimizu et al., 2018). Pouches can interfere with RFA, resulting in prolonged RFA duration (Chen et al., 2011; Shimizu et al., 2018). The sub-Eustachian pouch depth was independently associated with total RFA duration and radiofrequency energy delivered during cavotricuspid isthmus ablation (Shimizu et al., 2018). Some experts believe that resistive heating and impedance reduction at the beginning of RFA are associated with the amount of cardiac tissue surrounding the catheter tip (Viles-Gonzalez et al., 2017). When the catheter is placed in the pouch, its tip is surrounded by more cardiac tissue; therefore, a large amount of heat is rapidly generated. The rapid blood flow through the heart removes some of the heat, which is important to lower the temperatures of the catheter tip and cardiac tissue. However, blood flows slowly through the pouches. Therefore, theoretically, SP is more likely to occur in the pouches, but this assumption has not evaluated in previous studies.

Large pouches, such as those in the cavotricuspid isthmus and atrial septum, can be detected by intracardiac echocardiography, transesophageal echocardiography, intracardiac imaging, and multislice computed tomography (Shimizu et al., 2018). However, there is no effective, safe, and easily applicable method to detect small pouches, such as those in the left atrium.

To explore the relationships between pouch, impedance, and SP, we aimed to determine whether SPs occur more frequently in pouches, whether impedance differs between the pouch and the smooth myocardium, and whether impedance-related settings can be used to improve the safety of RFA.

## Materials and Methods

### Experimental Instruments

**Figure 2:**
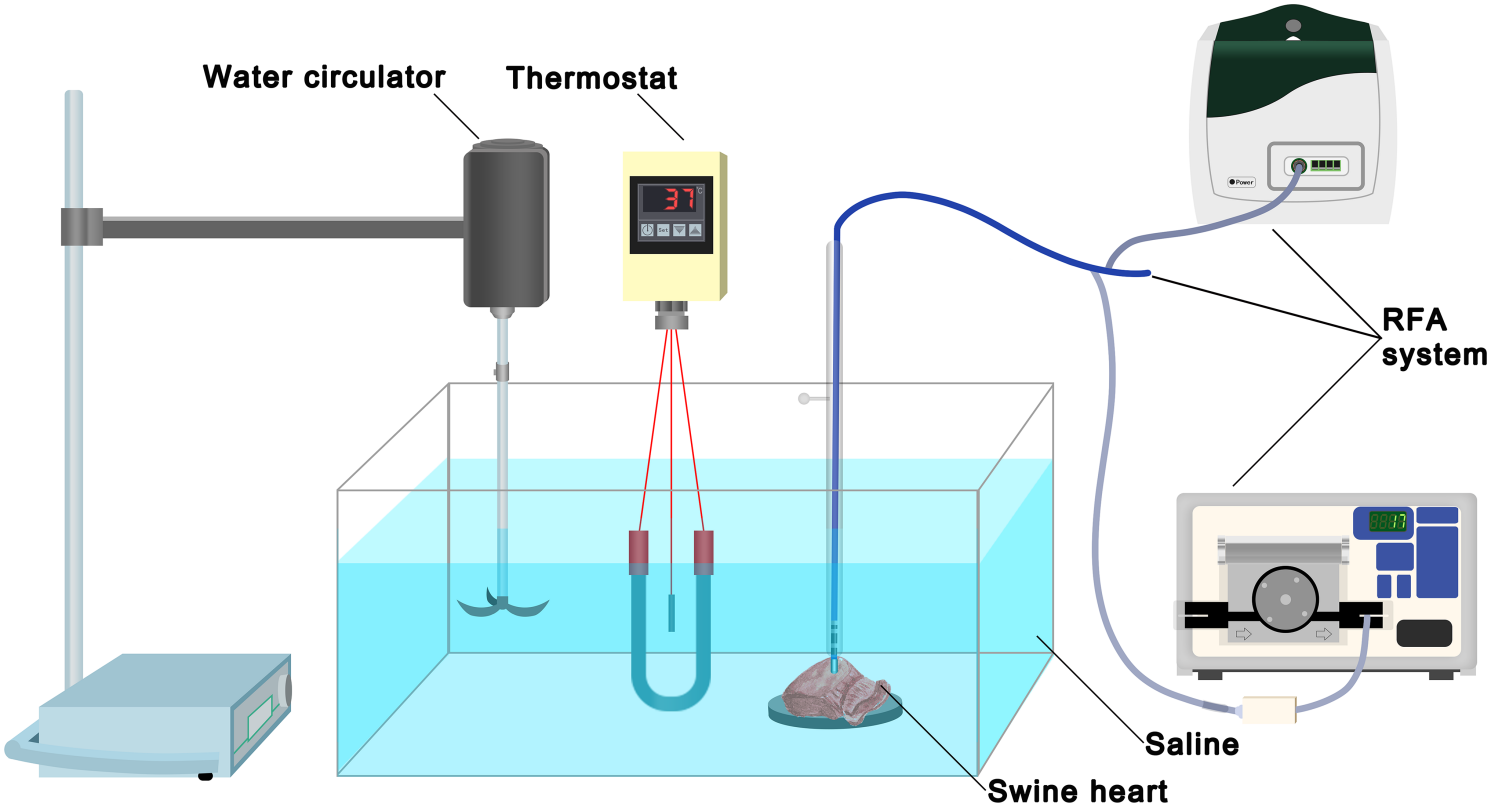
Experimental set-up. The experimental instruments consisted of a container with 0.9% saline, a thermostat, a water circulator, and an RFA system. The thermostat maintained the temperature of saline at 37°C and the water circulator continuously circulated the saline. Swine heart tissue was secured on a rubber plate in the saline container. RFA, radiofrequency ablation.

The experimental instruments consisted of a container with saline, a thermostat, a water circulator, and an RFA system (Figure 2). The thermostat maintained the temperature of the saline at 37°C, and the water circulator continuously circulated the saline. Swine hearts were obtained from commercial vendors and transported to the laboratory on ice immediately after the slaughter. The myocardium was cut into small pieces (5 × 5 cm), which were spread out and fixed on a rubber plate in saline. A total of 37 swine hearts were used.

The EnSite Precision system (St. Jude Medical Inc., St. Paul, MN, United States) and an Ampere^™^ radiofrequency generator were used for RFA. The TactiCath^™^ quartz contact force-sensing ablation catheter (75/65) was fixed in a plastic tube perpendicular to the tissue to maintain stability. The dynamic force sensor (TactiSys Quartz) was used to maintain a stable force (15–20 g) at the distal end of the catheter. The rate of irrigation flow of cold saline was 17 ml/min at the distal end of the catheter end (Cool Point Irrigation Pump).

### Experiment Design

Pouch and smooth myocardium were identified by observing the myocardial shape. Parts of the pouch and regular smooth myocardium co-existed in the same sections; multiple ablations were performed in the pouch and smooth myocardial areas on the same tissue sections.

The experiment comprised of four parts. First, continuous RFA at 50 W was applied until SP occurred. The RFA duration before SP was recorded for 40 RFAs performed in the pouch group and regular smooth myocardium group each. Second, impedance of the pouch group and smooth myocardial group without RFA was recorded. Third, continuous RFA at 50 W was applied to the pouch and smooth myocardial tissues, and impedance reduction within the first 3 s was recorded. The percentage of delta impedance (PDI, %) was calculated as (delta impedance/initial impedance) × 100. A total of 40 RFAs were performed in the pouch and smooth myocardium groups each. Fourth, continuous RFA at 50 W was applied using the delta impedance cutoff function (i.e., when delta impedance was higher or lower than the set value within the set delta time, the system automatically turned off the RFA). The following setting procedure was completed on the screen of the RF generator: Menu → Ablation Parameters → Delta Impedance Cutoff → Enable. For example, if delta impedance and delta time were set at 20 Ω and 3 s, respectively, the system will automatically terminate the RFA if the delta impedance reached 20 Ω within 3 s. Delta impedance was tested at a range of 1–50 Ω, and delta time was tested at a range of 1–10 s. Because the initial myocardial impedance varies between different locations, subgroups based on PDI are better able to reflect the changes in impedance. RFA was performed 40 times at each cutoff value in each group.

### Statistical Analysis

Normally distributed data are expressed as mean ± standard deviation (SD); data with a skewed distribution are expressed as median (interquartile range, IQR: P_25_–P_75_). A two-sample *t*-test was used to compare the data with normal distribution and homogeneity of variance. The Mann–Whitney *U*-test was used for data with a non-normal distribution. Categorical and grade data are expressed as number, rate, or percentage. The Kaplan–Meier method was used to analyze the probabilities of endpoint events. A time-event curve was constructed. A two-sided value of *p* < 0.05 was considered statistically significant.

## Results

### RFA Duration Before SP Was Shorter in Myocardial Pouches

**Figure 3:**
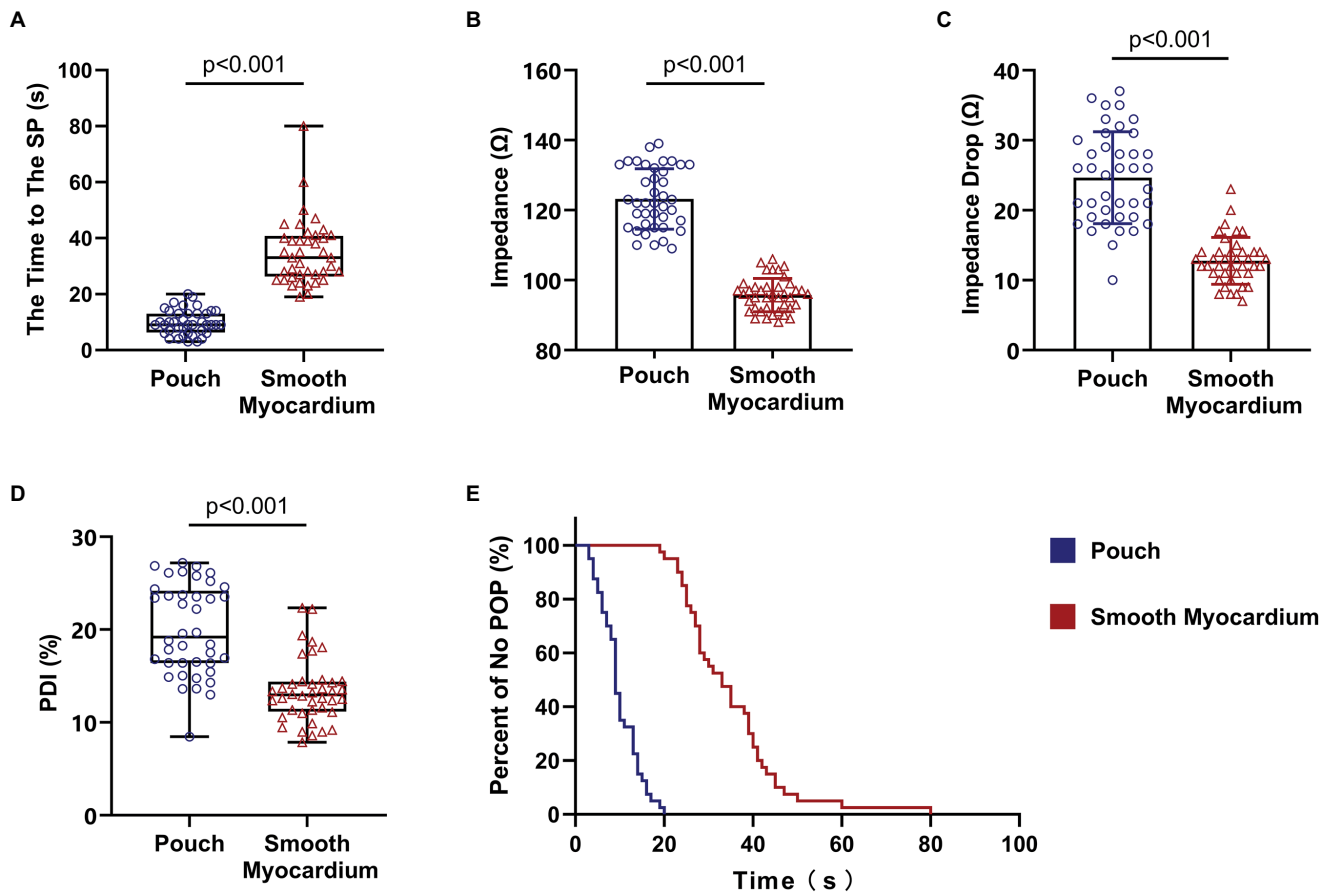
Steam pops occurred earlier in the pouch group. Impedance of the pouch tissue was high and rapidly decreased at the beginning of RFA. **(A)** RFA duration before SPs was shorter in the pouch group compared to the smooth myocardium group. **(B)** Impedance was higher in the pouch group compared to the smooth myocardium group. **(C)** Decrease in impedance in the first 3 s of RFA was faster in the pouch group compared to the smooth myocardium group. **(D)** The PDI of the pouch group was greater than that of the smooth myocardium group. **(E)** Kaplan–Meier curves show that SPs occurred earlier in the pouch group compared to the smooth myocardium group. PDI, percentage of delta impedance.

Continuous RFA at 50 W, without the cutoff function, caused SPs in all ablation lesions in the pouch and smooth myocardium groups. RFA duration before the SP was significantly shorter in the pouch group compared to the smooth myocardium group [9 s (IQR: 6.25–13 s) and 33 s (IQR: 26.25–40.75 s), respectively; *p* < 0.001; Figure 3A].

The Kaplan–Meier curves with SP as the endpoint event (Figure 3E) confirmed that SP occurred earlier in the pouch group compared to the smooth myocardium group.

### Impedance Change Was More Significant in Pouches Than Smooth Myocardium

**Figure 4:**
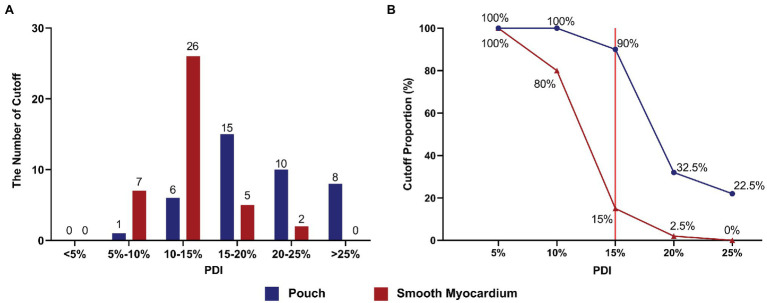
Appropriate PDI cutoff settings effectively prevented SPs. **(A)** The number of ablation lesions with PDI values (< 5%, 5–10%, 10–15%, 15–20%, 20–25%, and > 25%) during the first 3 s of RFA in the pouch group and smooth myocardium group. **(B)** The frequency of invoked cutoffs at different PDI settings. Red line indicates that at the optimal PDI setting of 15%, the cutoff rate in the pouch group was 90%, while RFA application was only terminated in 15% of all cases in the smooth myocardium group.

The initial impedance was significantly higher in the pouch group compared to the smooth myocardium group (123.22 ± 8.63 Ω and 95.75 ± 4.75 Ω, respectively; *p* < 0.001; Figure 3B). The impedance significantly decreased in the first 3 s of RFA at 50 W in both groups when the delta impedance cutoff function was disabled. The decrease in impedance was much greater in the pouch group compared to the smooth myocardium group (24.65 ± 6.57 Ω and 12.78 ± 3.35 Ω, respectively; *p* < 0.001; Figure 3C), confirming that the pouch areas had a more significant impedance change during RFA. Moreover, PDI was significantly higher in the pouch group compared to the smooth myocardium group [19.18% (IQR: 16.39–24.20%) and 12.96% (IQR: 11.17–14.39%), respectively; *p* < 0.001; Figure 3D]. PDI during the first 3 s of RFA was stratified as <5%, 5–10%, 10–15%, 15–20%, 20–25%, and > 25%. Among these PDI-based strata, 0, 1, 6, 15, 10, and 8 ablation lesions, respectively, were observed in the pouch group, and 0, 7, 26, 5, 2, and 0 ablation lesions, respectively, were observed in the smooth myocardium group (Figure 4A).

### SPs Are Prevented With the Use of Delta Impedance Cutoff

Using the delta impedance cutoff function, RFA procedure was continued until the cutoff was reached or SP occurred. RFA was performed using different PDI cutoff values (i.e., 5, 10, 15, 20, and 25%). For example, when a PDI cutoff value of 5% was selected and the delta impedance exceeded 5% of the initial impedance value, RFA was terminated.

The cutoff value was reached and RFA was terminated in some ablation lesions in both groups. No SP occurred in these cases. However, SP occurred in ablation lesions without the cutoff function. Using different PDI cutoff values, the rates of cutoff without SP were 100 and 100% (PDI = 5%), 100 and 80% (PDI = 10%), 90 and 15% (PDI = 15%), 32.5 and 2.5% (PDI = 20%), and 22.5 and 0% (PDI = 25%) in the pouch group and smooth myocardium group, respectively (Figure 4B).

**Figure 5:**
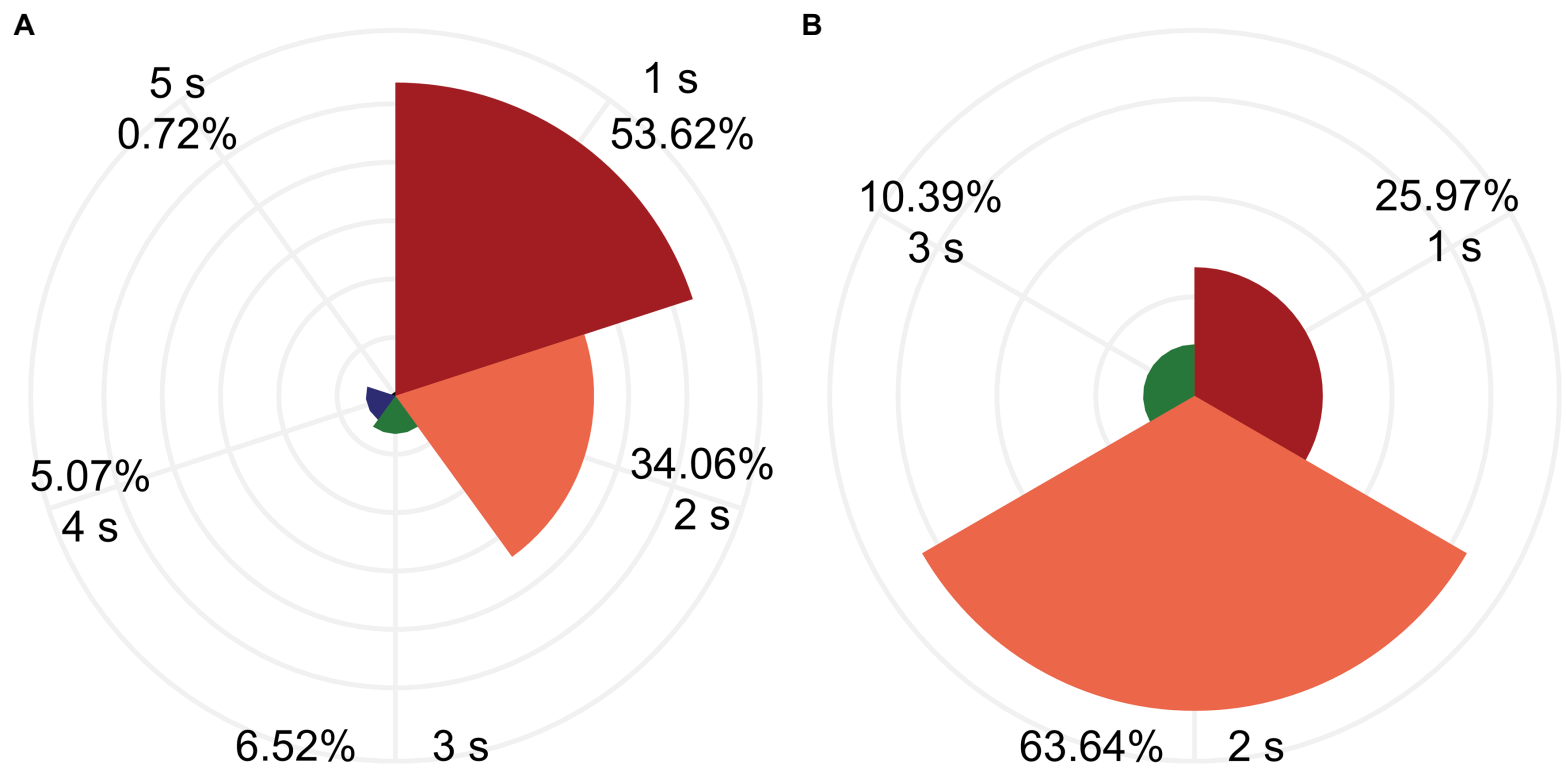
Most cutoffs occurred within the first 3 s of RFA. RFA duration before the cutoff was 1–5 s. Percentage of RFA duration in the pouch group **(A)** and smooth myocardial group **(B)** are depicted.

Because five cutoff threshold values were set, 200 RFAs were performed (5 thresholds × 40 lesions) in each group. Cutoff was invoked in 138 and 77 RFAs in the pouch group and smooth myocardium group, respectively. The RFA duration before cutoff was 1–5 s (minimum to maximum). In the pouch group, most cutoffs occurred within 1–3 s after the start of RFA (130/138 cutoffs, 94.20%), and a few cutoffs occurred in 4 s (7/138, 5.07%) and 5 s (1/138, 0.72%). In the smooth myocardium group, all cutoffs occurred within the initial 3 s (77/77 cutoffs, 100%; Figure 5), confirming that significant impedance changes occurred at the beginning of RFA.

## Discussion

### SPs Occur More Frequently and Earlier in Pouch Areas

When the catheter tip was placed in a pouch, it was surrounded by more myocardial tissue compared to when it was placed on smooth myocardium, producing a greater amount of heat (Viles-Gonzalez et al., 2017). In addition, less blood flow passes through the pouch to remove the heat. Therefore, heat builds up much faster in pouches compared to the smooth myocardium, causing SPs during RFA. Because the presence of pouches is a risk factor of SP, techniques to detect the presence of pouches or avoid RFA in the pouches will reduce the incidences of SP and other serious complications. Such techniques are especially desirable for high-power, short-time RFA, which involve a rapid increase in temperature and strictly controlled ablation.

In our study, the pouch group and smooth myocardium group were separated based on visual inspection. The characteristics of the pouches that may increase the incidence of SP are as: firstly, the diameter of the pouch is larger than the outer diameter of the ablation catheter so that the catheter can fall into and ablate within the pouch. Currently, the outer diameter of the ablation catheter used commonly is greater than 7F (2.331 mm), so pouch should be greater than 2.331 mm in diameter. Secondly, a shallower and greater pouch is more conducive to heat dissipation and less likely to produce SP.

### Impedance of Pouch Myocardium Is Significantly Increased

It is not completely clear why impedance is higher in pouches compared to the smooth myocardium. Marmar et al. suggested that the main determinant of impedance is the cross-sectional area of the lumen where the catheter tip is placed (Vaseghi et al., 2005). The cross-sectional area of a pouch is significantly smaller than that of the smooth myocardium. Therefore, the pouch generally has higher impedance. In addition, blood is an excellent electronic conduction medium, and abundant blood flow decreases the impedance of the myocardial tissue. Pouch cavities in the left atrial/ventricle endocardium have significantly reduced blood flow due to their small size, while the smooth myocardium is rich in blood flow, which increases the difference in the impedance of the two myocardial regions. Cheung et al. (2004) and Vaseghi et al. (2005) reported that the pulmonary veins have relatively high impedance (Cheung et al., 2004; Vaseghi et al., 2005), which may complicate pulmonary vein RFA by causing pulmonary vein stenosis or even occlusion (Teunissen et al., 2017; Fink et al., 2018). In this study, RFA in the pouch was associated with increased risk of SP. Therefore, RFA-related risks are increased in areas with high impedance, such as pouch and the pulmonary vein. A significant increase in impedance during the movement of the catheter suggests that the catheter tip may have entered a special anatomical site, such as a pouch or pulmonary vein. Therefore, in such a case, the catheter should be repositioned until the impedance decreases to the initial level, and RFA can be resumed.

Using the delta impedance cutoff function, cutoff will be invoked when the impedance increases or decreases to a certain value. In China, most RFA procedures are performed under local anesthesia rather than general anesthesia; therefore, the catheter tip is prone to movements during the operation due to unstable breathing, coughing, and body movements. Large abnormal movements of the catheter tip during RFA, such as sliding into the pulmonary vein from the atrial wall, may lead to dramatic impedance changes, which will invoke cutoff and automatically terminate the RFA. The cutoff method can reduce the likelihood of performing ablation under unstable conditions and improve RFA efficiency and safety. However, the optimal cutoff value has not been determined.

In clinical practice, SP is often accompanied by significant changes in impedance. When designing the study protocol, we tried to reduce SP by interfering with this impedance change. However, we gave up finally, because it was found that the interval between these impedance change and SP was very short or even simultaneous, so it was difficult to intervene by impedance cutting. This impedance change may be related to tissue change or catheter displacement during SP occurrence.

### Appropriate Cutoff Settings Can Prevent SPs and Ensure Normal RFA Application

During the first 3 s of RFA, the impedance significantly decreased in the pouch group and smooth myocardium group; both the impedance reduction and PDI were greater in the pouch group compared to the smooth myocardium group. A low PDI threshold easily invoked cutoff, thereby preventing SPs and interfering with the normal RFA procedures. Conversely, when a high PDI threshold was used, cutoff was not frequently invoked, thereby rarely interfering with the normal RFA procedures. However, the incidence of SP increased. When the PDI threshold was set to 15% (red reference line in Figure 4B) and delta time to 3 s, 90% of RFAs in the pouch group were cutoff, while only 15% of RFAs were terminated in the smooth myocardium group (Figure 4B). Therefore, PDI of 15% and delta time of 3 s are the optimal cutoff settings, which significantly reduce the incidence of SPs, while having minimal effect on normal RFAs.

In this study, we observed that the impedance decreased rapidly in the initial stage of continuous RFA and decreased slowly in the later stage, consistent with the previous studies (Nakagawa et al., 1998). In the present study, 96.28% (207/215) of the cutoffs occurred within the first 3 s of RFA, confirming a significant decrease in impedance in the initial stage. At 50 W, the RFA durations before SP were 9 s (IQR: 6.25–13 s) and 33 s (IQR: 26.25–40.75 s) in the pouch group and the smooth myocardium group, respectively. Both durations were significantly longer than the 3 s required to invoke cutoffs. Therefore, cutoffs usually occurred before SPs, thereby lowering SP incidence and ensuring RFA safety.

### Study Limitations

The ablation power we used was 50 W, and the results were obtained under experimental conditions. In clinical practice, parameters may vary according to different conditions. When designing the study protocol, we attempted to conduct a study *in vivo*. However, in this study, the pouch group and smooth myocardium group were separated based on visual inspection, which is difficult to do *in vivo*. Temperature is associated with SP production. In order to simulate temperature changes in the real environment, myocardial tissue needs to be immersed in a large amount of flowing fluid during RFA. We also tried to replace saline with heparinized blood. However, we found that pouch was difficult to visualize in this blood, so we did not use heparinized blood in place of saline in our model ultimately. Therefore, further research is needed to identify the optimal cutoffs settings for RFA in human tissue. With advancements in catheter-based RFA technology, it is likely that no single factor will be able to predict the occurrence of SPs. Therefore, a combination of parameters may be evaluated to predict and prevent SP formation.

## Conclusion

The endocardial pouch structure has a higher initial impedance and a faster impedance decreases during RFA. The endocardial pouch structure is prone to the occurrence of SPs. The use of an appropriate delta impedance cutoff threshold (PDI of 15% and delta time of 3 s) can effectively prevent SPs without affecting RFA application, thereby increasing RFA safety.

## Data Availability Statement

The raw data supporting the conclusions of this article will be made available by the authors, without undue reservation.

## Ethics Statement

Ethical review and approval was not required for the animal study because Swine hearts were obtained from commercial vendors.

## Author Contributions

JL, JX, and FG conceived and designed the animal experiments. HZ, HS, and YW contributed to the statistical analysis. JX, JL, JZ, and CZ wrote the main body of the manuscript. All authors contributed to the critical reading and approval of the manuscript.

## Funding

This work was supported by the Central Government Guidance for Local Science and Technology Development (2017070802D145).

## Conflict of Interest

The authors declare that the research was conducted in the absence of any commercial or financial relationships that could be construed as a potential conflict of interest.

## Publisher’s Note

All claims expressed in this article are solely those of the authors and do not necessarily represent those of their affiliated organizations, or those of the publisher, the editors and the reviewers. Any product that may be evaluated in this article, or claim that may be made by its manufacturer, is not guaranteed or endorsed by the publisher.
